# Characterization of tryptanthrin as an antibacterial reagent inhibiting *Vibrio splendidus*

**DOI:** 10.1007/s00253-024-13158-7

**Published:** 2024-05-24

**Authors:** Huirong Yang, Ya Li, Weibo Shi, Weiwei Zhang

**Affiliations:** 1 Collaborative Innovation Center for Zhejiang Marine High-Efficiency and Healthy Aquaculture, Ningbo University, Ningbo, 315832 People’s Republic of China; 2 School of Marine Sciences, Ningbo University, 169 Qixingnan Road, Ningbo, Zhejiang Province 315832 People’s Republic of China

**Keywords:** *Vibrio splendidus*, Tryptanthrin, Bactericide, LuxO

## Abstract

**Abstract:**

Isolates of *Vibrio splendidus* are ubiquitously presented in various marine environments, and they can infect diverse marine culture animals, leading to high mortality and economic loss. Therefore, a control strategy of the infection caused by *V. splendidus* is urgently recommended. Tryptanthrin is a naturally extracted bioactive chemical with antimicrobial activity to other bacteria. In this study, the effects of tryptanthrin on the bacterial growth and virulence-related factors of one pathogenic strain *V. splendidus* AJ01 were determined. Tryptanthrin (10 μg/mL) could completely inhibit the growth of *V. splendidus* AJ01. The virulence-related factors of *V. splendidus* AJ01 were affected in the presence of tryptanthrin. Tryptanthrin resulted an increase in biofilm formation, but lead to reduction in the motility and hemolytic activity of *V. splendidus* cells. In the cells treated with tryptanthrin, two distinctly differentially expressed extracellular proteins, proteases and flagellum, were identified using SDS**–**PAGE combined with LC**–**MS. Real-time reverse transcriptase PCR confirmed that the genes involved in the flagellar formation and hemolysin decreased, whereas specific extracellular proteases and the genes involved in the biofilm formation were upregulated. Two previously annotated *luxO*_*Vs*_ genes were cloned, and their expression levels were analyzed at different cell densities. Molecular docking was performed to predict the interaction between LuxO_Vs_ and ATP/tryptanthrin. The two sigma-54-dependent transcriptional regulators showed similar ATP or tryptanthrin binding capacity but with different sites, and the direct competitive binding between ATP and tryptanthrin was present only in their binding to LuxO_1_. These results indicated that tryptanthrin can be used as a bactericide of *V. splendidus* by inhibiting the growth, bacterial flagella, and extracellular proteases, but increasing the biofilm. Sigma-54-dependent transcriptional regulator, especially the quorum sensing regulatory protein LuxO_1_, was determined to be the potential target of tryptanthrin.

**Key points:**

• *Tryptanthrin inhibited the growth of V. splendidus in a dose-dependent manner.*

• *The effect of tryptanthrin on the virulence factors of V. splendidus was characterized.*

• *LuxO was the potential target for tryptanthrin based on molecular docking.*

**Supplementary Information:**

The online version contains supplementary material available at 10.1007/s00253-024-13158-7.

## Introduction

*Vibrio splendidus* is an important opportunistic pathogen, and isolates belonging to this species are ubiquitously present in seawater and sediments. They could infect a wide range of cultured animals from fish to invertebrate (Zhang and Li 2021). The infection of *V. splendidus* could lead to various diseases, resulting in mass mortalities among shellfish (Le Roux et al. 2004) and fish (Jensen et al. 2003). In particular, *V. splendidus* could infect the highly valued sea cucumber *Apostichopus japonicus* and result in disease with skin ulcer syndrome. Bacterial swimming, biofilm formation, and hemolysin are the virulence-related factors of *V. splendidus* (Zhang and Li 2021; Li et al. 2023; Yang et al. 2023).

Antibiotics are the most frequently and commonly used agents to cope with bacterial infection (Han et al. 2016). For example, furan and quinolones have been used to cure sea cucumber *A. japonicus* suffering from totting edge symptoms at the auricularia stage and the gas bubble–diseased sea cucumber *A. japonicus* suffering at the stage of auricularia due to *Vibrio* sp. (Han et al. 2016). However, the remaining antibiotics in the aquatic environment can cause severe environmental problems. The residue of antibiotics can frequently generate and spread antibiotic resistances (Polianciuc et al. 2020). Therefore, new environmentally friendly chemicals to substitute antibiotics have become the promising tools to prevent *Vibrio* sp. infection (Wang et al. 2017). One representative kind of these chemicals is the quorum sensing inhibitors, and coumarin targeting the quorum sensing regulation has been used for the control of *V. splendidus* infection (Zhang et al. 2017). Pyoverdine interfering the iron uptake process was determined to be effective in controlling *V. splendidus* infection (Zhang et al. 2016).

Extracting biochemical agents from Chinese traditional plants is an effective method to obtain new antimicrobial agents to inhibit the biomass or the virulence of bacterial pathogens (Pu et al. 2020). Tryptanthrin is a natural alkaloidal compound with the chemical name 6,12-dihydro-6,12-dioxoindolo-(2,1-b)-quinazoline, and it is distributed worldwide (Bigoniya and Rana 2009; Kawakami et al. 2011). The therapeutic effect of tryptanthrin has been confirmed to inhibit the expression of matrix metalloproteinase (MMP)-3 gene in different cell lines (Kirpotina et al. 2020). Tryptanthrin also possesses the antituberculotic activity, antiprotozoal activity, antioxidant, and antimicrobial activities (Kaur et al. 2017). As for the antimicrobial activity, tryptanthrin from the leaves of *Strobilanthes cusia* can inhibit the growth of *Trichophyton mentagrophytes* (Honda et al. 1979). Recently, tryptanthrin has also been used to strongly inhibit the biofilm formation activity of *Vibrio cholerae* (Narendrakumar et al. 2019).

In the present study, the growth and virulence-related factors of *V. splendidus* AJ01 by tryptanthrin were determined. The biofilm formation, hemolytic activity, extracellular protein secretion, and bacterial motility of *V. splendidus* AJ01 were characterized. Molecular docking between LuxO and tryptanthrin was performed to detect whether LuxO could be the target of tryptanthrin in *V. splendidus*. This study offers a new antibacterial chemical to treat *V. splendidus* infection in the future.

## Materials and methods

### Bacteria, culture, and regents

*V. splendidus* AJ01 was stocked in our lab and was preserved with a name Vs in CGMCC with an accession number of 7.242. *V. splendidus* AJ01 was cultured in 2216E medium, which was prepared with 1-g yeast extract, 5 g tryptone, and 0.01 g FePO_4_ in 1-L aged seawater. The bacterial culture was incubated in one shaker with speeds of 120–150 rpm/min (Jiangnan Instrument Co., Ningbo, China). Tryptanthrin was bought from Shanghai Yuanye Biotechnology Co. (CAS 13220–57-0, Shanghai, China), and it was dissolved into dimethyl sulfoxide (DMSO) to make a stock solution of 2.5 mg/mL. The other chemicals were bought from Shanghai Sangon Co. (China), unless otherwise stated.

### Molecular technology

Through searching the genomic DNA of AJ01, two genes annotated to code LuxO repressor protein were selected. Both were amplified by PCR using two pairs of primers of *luxO*_*1*_-F/*luxO*_*1*_-R and *luxO*_*2*_-F/*luxO*_*2*_-R (Table 1), respectively. The sequences of the *luxO*_*1*_ and *luxO*_*2*_ genes were sequenced by Sangon (Shanghai, China) and verified by BLAST software in the website of the National Centre for Biotechnology Information (http://www.ncbi.nlm.nih.gov/blast). The expert protein analysis system (http://www.expasy.org/) was used to determine the molecular mass, amino acid sequence, and theoretical isoelectric point (pI). SMART was used to analyze the domains (http://smart.embl.de/). The three-dimensional structure of LuxO_1_ and LuxO_2_ was constructed using the SWISS-MODEL (https://web.expasy.org/protparam/). Molecular phylogenetic tree was constructed using MEGA7.0 software with neighbor-joining tree method.

**Table 1:** The primers used in this study

Primer name	Sequence (5′ → 3′)
LuxO_1_F	GGATCCATGGTAGAGGATACCGCTTCG
LuxO_1_R	GCGGCCGCTTACTGCTTCGCATTCCACGT
LuxO_2_F	GGATCCATGCGCCCTAAAGTATTGTTG
LuxO_2_R	GCGGCCGCTTAGGCTTGATTGTACTCATC
933F	GCACAAGCGGTGGAGCATGTGG
16SRTR	CGTGTGTAGCCCTGGTCGTA
flhG F	GCGGGTGAGTGTGAACTTAAGGA
flhG R	AACGCTCGAATCAAACCAGGATG
fliE F	CACTACAAAAGACCTCTGGCGAT
fliE R	CACACTAGATTTATTCCGAGCGA
flhB F	CTGCTTATTAGTTGCTCTTTGTT
flhB R	TTCAGGCTTACCTTCAGTGTCTT
flgG F	TCAAACAACCTTGCCAACGCCTC
flgG R	ACCAGCACCCAACATCAAACCAC
flgA F	TTTAGACGCCCAACAAACCAATA
flgA R	ACCAGCACCCAACATCAAACCAC
motY F	TCGCAATGTCAGTTTGATTTCTA
motY R	AATACCCCAAGCTGTTTGTCCCC
AD F	AAGTTATTGGCTGTGGAAGGAAA
AD R	TCGGCGAGTATGGAGACTGGTGA
hlyI F	GGCTGTATTTGCCGTTGTATTAG
hlyI R	GATTGTTTTCAGTCACCGTGTCT
hlyII F	ACCGAACTGACGCATCACATGAT
hlyII R	TTTGAAAGAGACGGAAGACCACC
hlyIII F	TGGCTTAGGCGTAGTGCTTGGTG
hlyIII R	GCGCTTGGTTTTTTCTGTGGTGA
ZP1 F	CAGTGTGCCAAGTGTTTCTGCAC
ZP1 R	ATCGAACTCCTCTCCTAGTTCCG
ZP2 F	AATGACCTAGAAGACAACCCACA
ZP2 R	GAATGAATACTCACCAGCAAAAG
LuxO_1_F	TCTTACACCGCTCGAGATTGATAT
LuxO_1_R	TAAAAATAACTGGAACTTCTGGGT
LuxO_2_F	TCAAATCTATCGACAAAGAGACACA
LuxO_2_R	TGATTGTACTCATCTTCAGATTCCC

### Measurement of minimal inhibitory concentration (MIC)

MIC of tryptanthrin was determined as described previously (Narendrakumar et al. 2019). Briefly, 2216E media supplemented with 0.5, 1, 2.5, 5, 10, 25, and 50 μg/mL tryptanthrin were used to culture *V. splendidus* AJ01. The control sample was the culture of *V. splendidus* AJ01 grown in medium without tryptanthrin. After being cultured for 24 h, OD_600_ was recorded using a microplate reader (FlexA-200, Allsheng, China). Each growth was performed and measured in triplicate.

### Biofilm measurement

Biofilm was determined as previous description (O’Toole 2011) with minor modification. *V. splendidus* AJ01 was reinoculated into fresh 2216E medium to make an initial bacterial cell suspension of 1.0 × 10^6^ CFU/mL. The cell suspension was split into a 96-well polystyrene microplate. 2.5 μg/mL tryptanthrin was added as the experimental group, and the culture with the same volume of DMSO was used as the control sample. The plates were statically cultured in an incubator at 28 ℃ for 36 h, and then, the OD_600_ of the culture was measured. After that, biofilm was stained using crystal violet, and the absorbance at OD_590_ using a spectrophotometer (FlexA-200, Allsheng, China).

### Swimming test

Swimming motility of *V. splendidus* AJ01 was performed according to the previous method (Wang et al. 2023). *V. splendidus* AJ01 was grown in medium supplemented with 2.5 μg/mL tryptanthrin for 24 h; then, 10-μL bacterial culture was plugged into the swimming agar, i.e., 2216E medium containing 2.5 μg/mL of tryptanthrin and 0.3% agar. The cells grown without tryptanthrin in both liquid medium and swimming agar were used as control. Diameter of the swimming circle was measured after inoculated for 24 h. Each experiment was carried out in triplicate.

### Hemolytic activity assay

Defatted sheep blood was used to detect the hemolytic activity of *V. splendidus* AJ01 according to the description by De et al. (1954). Briefly, *V. splendidus* AJ01 was grown with 2.5 μg/mL tryptanthrin for 24 h; then, 10-μL bacterial culture was separately dropped onto the solid culture medium simultaneously containing 5% sheep blood cells and 2.5 μg/mL tryptanthrin. The cells grown without tryptanthrin in both liquid medium and agar were used as control. Then, all these plates were left statically at 28 ℃ for 24 h, and the diameter of hemolytic circle with or without tryptanthrin was measured.

### Extraction of extracellular proteins

Overnight culture of *V. splendidus* AJ01 was reinoculated into 2216E medium at a volume of 1%. 2.5 μg/mL tryptanthrin was added and equal volume of DMSO was added as a control. The cultures were grown to a OD_600_ value of approximately 0.4, and then, the supernatants were collected after centrifugation, followed by filtration through a 0.22 μM membrane (Millipore, the USA) to obtain the cell-free supernatant. Then, 1/6 volume of 10% TCA was added. The mixture was left at 4 ℃ for 30 min, followed by centrifugation at 10,000 × g for 10 min to pellet the extracellular proteins. The precipitate was finally dissolved in buffer B (100 mM NaH_2_PO_4_ and 8 M urea in 10 mM Tris–Cl).

### SDS–PAGE and LC–MS

Twenty microliters of the sample was taken and mixed with 5 μL SDS**–**PAGE loading buffer, and the samples were boiled at 100 ℃ for 5 min. Then, the differentially expressed proteins (DEPs) in the extracellular proteins collected from the cultures grown with and without tryptanthrin were detected using SDS**–**PAGE. The bands with DEPs were cut off and smashed into about 1-mm^3^ small pieces. Finally, the proteins in the gels were analyzed using LC–MS by Sangon Co. (Shanghai, China).

### Scanning electron microscopy (SEM)

Bacterial cells were obtained by centrifugation of the cell culture at 3000 rpm at 4 ℃ for 2 min, and then, the cells were washed in triplicate in 100 mM PBS (pH 7.2–7.4). The cells were mixed with glutaraldehyde (2.5%) for 12 h, followed by washes with PBS, twice with 10 min for each time, and another twice washes with pure water. The sample was subsequently dehydrated with ethanol solution at gradients ranging from 30 to 90% for 15 min and finally dehydrated twice with pure ethanol, 15 min for each time. The samples were then resuspended in the mixture of tert-butanol and ethanol (1:1, v/v) for 15 min, followed by suspension in tert-butanol for another 15 min for twice. The samples were placed on 5 × 5 mm cover slips, frozen at − 80 °C, then put into a freeze dryer, and finally were observed under Hitachi SU8100 SEM (Japan).

### Real-time reverse transcriptase PCR (RT–PCR)

Real-time RT–PCR was used to determine the mRNA level of specific genes in the cells grown with tryptanthrin, when compared to their expression in the cells grown without tryptanthrin. One biofilm formation–related gene, six genes involved in the flagellar biosynthesis, and three hemolysins and *luxO* genes were chosen. To collect samples for quantitative real-time RT**–**PCR, overnight culture of *V. splendidus* AJ01 was inoculated into media with or without 2.5 μg/mL tryptanthrin, respectively. The cultures were grown to OD_600_ value of approximately 0.4; then, the cultures were centrifuged to collect the cell pellet. TRIzol was used to extract RNA, and ABclonal reverse transcription kit (ABclonal Technology Co., Wuhan, China) was used to synthesize cDNA. The primers in the real-time RT–PCR for each gene are listed in Table 1. Real-time RT**–**PCR experiment was performed in the ABI 7500 real-time system (Applied Biosystems, the USA) based on the SYBR ExScript RT**–**PCR kit (Takara, China). The experiment was carried out in triplicate using 16S rRNA as a control gene. Dissociation analysis of the PCR products was carried out at the end of each PCR, which was used to verification that only one DNA product was successfully amplified. The comparative 2^**–△△**CT^ method was occupied to compare the mRNA levels between different samples.

### Molecular docking analysis

LuxO was the target of tryptanthrin in *V. cholerae* according to the report of Boyaci et al. (2016). Considering the conserved sequence of LuxO in *Vibrio* sp., the interaction between tryptanthrin and LuxO_Vs_ in *V. splendidus* was determined by molecular docking using the software AutoDock v4.2. Three-dimensional structure of tryptanthrin was retrieved from the data in PubChem (http://pubchem.ncbi.nlm.nih.gov). PyMOL and AutoDock were used to detect whether tryptanthrin, as well as ATP, could bind to LuxO.

### Nucleotide sequence accession numbers

The nucleotide sequences have been deposited in the GenBank in NCBI. The nucleotide sequences of *flhG*, *fliE*, *flgG*, *flhB*, *flgA*, *motY*, *AD*, *ZP1*, *ZP2*, *hemolysin* I, *hemolysin* II, *hemolysin* III, *luxO*_1_, and *luxO*_2_ are under accession numbers OR743447, OR743448, OR743449, OR743450, OR743451, OR743452, OR743454, OR743455, OR743456, OR743457, OR743458, OR743459, OR743460, and OR799617, respectively.

## Result

### Tryptanthrin inhibited the growth and affected the cell morphology of *V. splendidus* AJ01

**Fig. 1:**
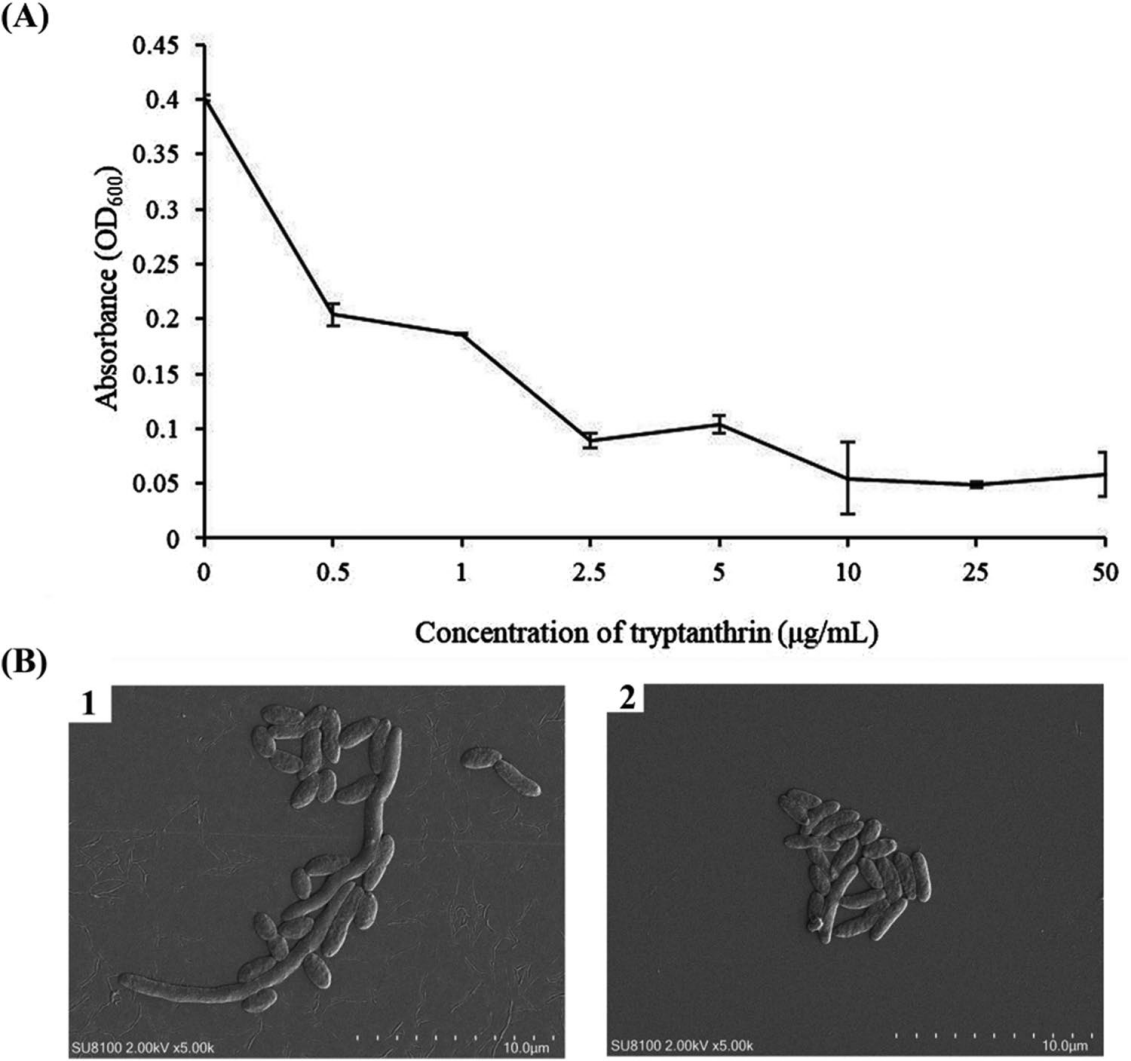
Effects of different concentrations of tryptanthrin on growth and morphology of *V. splendidus* AJ01. **A** OD_600_ of *V. splendidus* AJ01 culture grown with tryptanthrin. *V. splendidus* AJ01 was grown in 2216E media supplemented with 0, 0.5, 1, 2.5, 5, 10, 25, and 50 μg/mL tryptanthrin. The values are the mean ± standard errors from at least three experiments. **B** The representative figure of *V. splendidus* AJ01 cells grown with and without tryptanthrin, respectively, and morphology of *V. splendidus* AJ01 cells was observed under SEM. 1 represented *V. splendidus* cells without tryptanthrin; 2 represented *V. splendidus* cells with tryptanthrin

To test whether tryptanthrin could inhibit the growth of *V. splendidus* AJ01, an overnight culture of *V. splendidus* was inoculated into media with different concentrations of tryptanthrin. The results showed that *V. splendidus* AJ01 was highly sensitive to tryptanthrin, 80% of the growth of *V. splendidus* AJ01 could be reduced by 2.5 μg/mL tryptanthrin, and 10 μg/mL tryptanthrin completely inhibited the growth. Thus, 10 μg/mL was determined to be the MIC of tryptanthrin for the growth of *V. splendidus* AJ01 (Fig. 1**A**).

Tryptanthrin showed an obvious effect on the morphology of *V. splendidus* AJ01 observed under SEM. The untreated cells were intact, with vigorous growth and propagation. The long rod-shaped bacterial cells were the active cells that could divide into normal cells. After treatment with 2.5 μg/mL tryptanthrin, the cells became shorter, without the existence of extremely long rod-shaped bacterial cells. The cells also tended to cluster tightly. These results indicated that tryptanthrin could alter the morphology of bacterial cells, further interfering with their growth and survival (Fig. 1**B**).

### Tryptanthrin affected virulence-related factors

#### Tryptanthrin reduced the biofilm formation

**Fig. 2:**
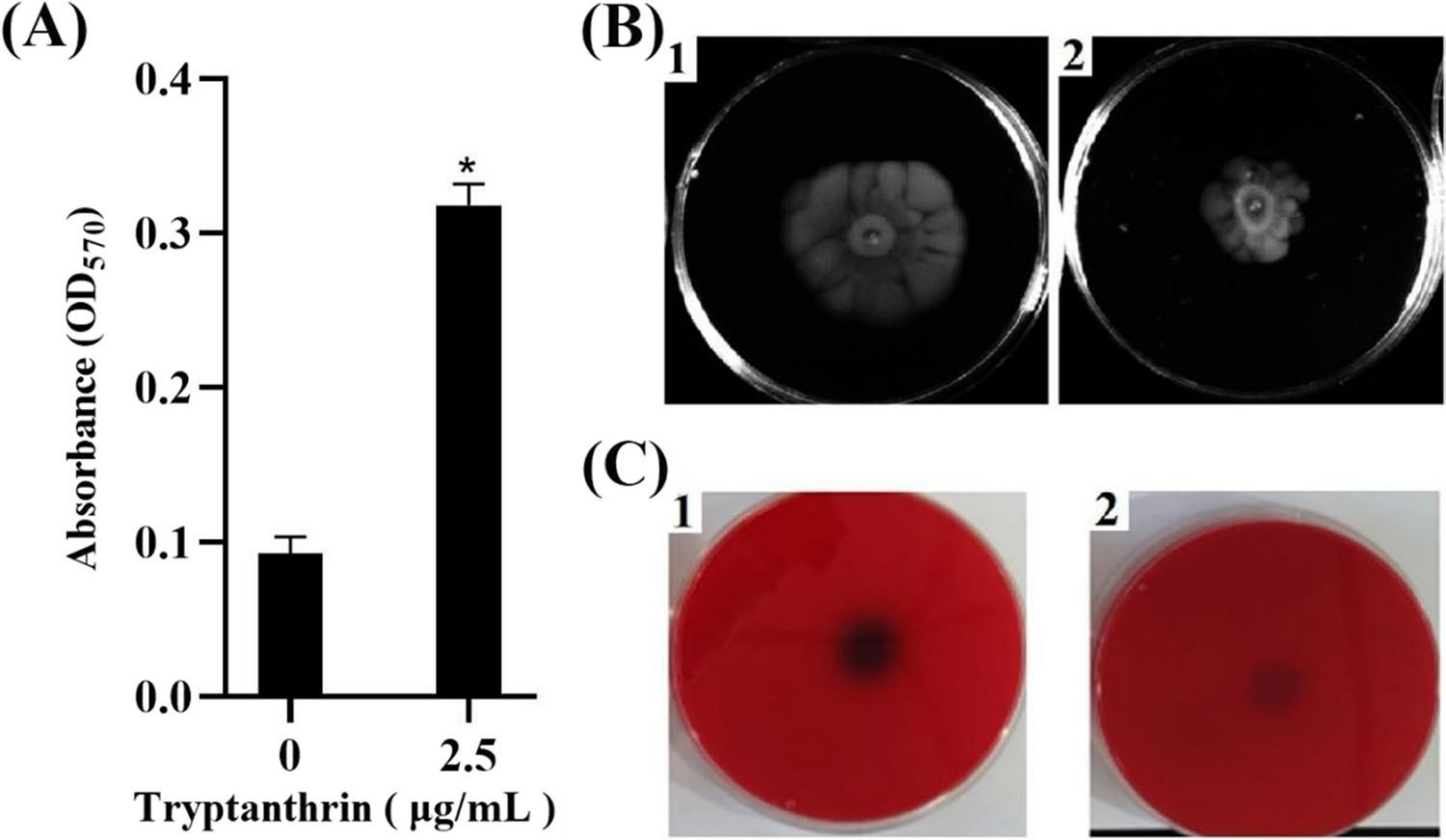
Determination of the virulence-related factors of *V. splendidus* AJ01 with tryptanthrin. **A** Quantitative measurement of the biofilm formed by *V. splendidus* AJ01 with tryptanthrin. *V. splendidus* AJ01 was grown in the presence or absence of tryptanthrin in 96-well plates for 24 h. Biofilm cells were stained with crystal violet, then dissolved using glacial acetic acid, and finally, OD_590_ was measured. Each growth was carried out in triplicate. *P < 0.05. **B** Swimming motility of *V. splendidus* AJ01 with tryptanthrin. Five microliters of culture with OD_600_ of approximately 0.4 with or without tryptanthrin was inserted into the center of the swimming agar. After being incubated for 24 h, the diameter of swimming circle was recorded. 1, the control cells without tryptanthrin; 2, the cells with tryptanthrin. **C** Hemolytic activity of *V. splendidus* AJ01 with or without tryptanthrin. Five-microliter culture of *V. splendidus* AJ01 (OD_600_ = 0.4) in the presence and absence of tryptanthrin was dropped onto the agar with sheep defatted blood. After being incubated at 28 ℃ for 24 h, the color was observed. 1, the control cells; 2, the cells with tryptanthrin. The experiment was performed in triplicate

Biofilm is an important virulence factor related to bacterial virulence, so the effect of tryptanthrin on the biofilm formation of *V. splendidus* was determined. After the cells were incubated with tryptanthrin for 36 h statically, the inhibitory effect of tryptanthrin was not as high as that in the liquid culture. The OD_600_ values of the cultures with and without 2.5 μg/mL tryptanthrin were 0.62, and 0.45, respectively. Accompanied with a reduction in biomass in the presence of tryptanthrin, the biomass of biofilm was higher than that without tryptanthrin (Fig. 2**A**). However, the swimming ability of AJ01 reduced in the cells grown with tryptanthrin. After being incubated for 24 h, the diameters of the *V. splendidus* AJ01 colony without and with tryptanthrin were approximately 30 mm and 15 mm, respectively (Fig. 2**B**).

#### Tryptanthrin inhibited the hemolytic activity of *V. splendidus*

Considering hemolysin is another important virulence factor of pathogenic bacteria, the effect of tryptanthrin on the hemolytic activity was determined. When defatted sheep blood was used, *V. splendidus* AJ01 exhibited a distinct α-hemolytic activity. The hemolytic activity of *V. splendidus* AJ01 in the presence of tryptanthrin significantly reduced (Fig. 2**C**).

#### Tryptanthrin changed the expression profiles of extracellular proteins

**Fig. 3:**
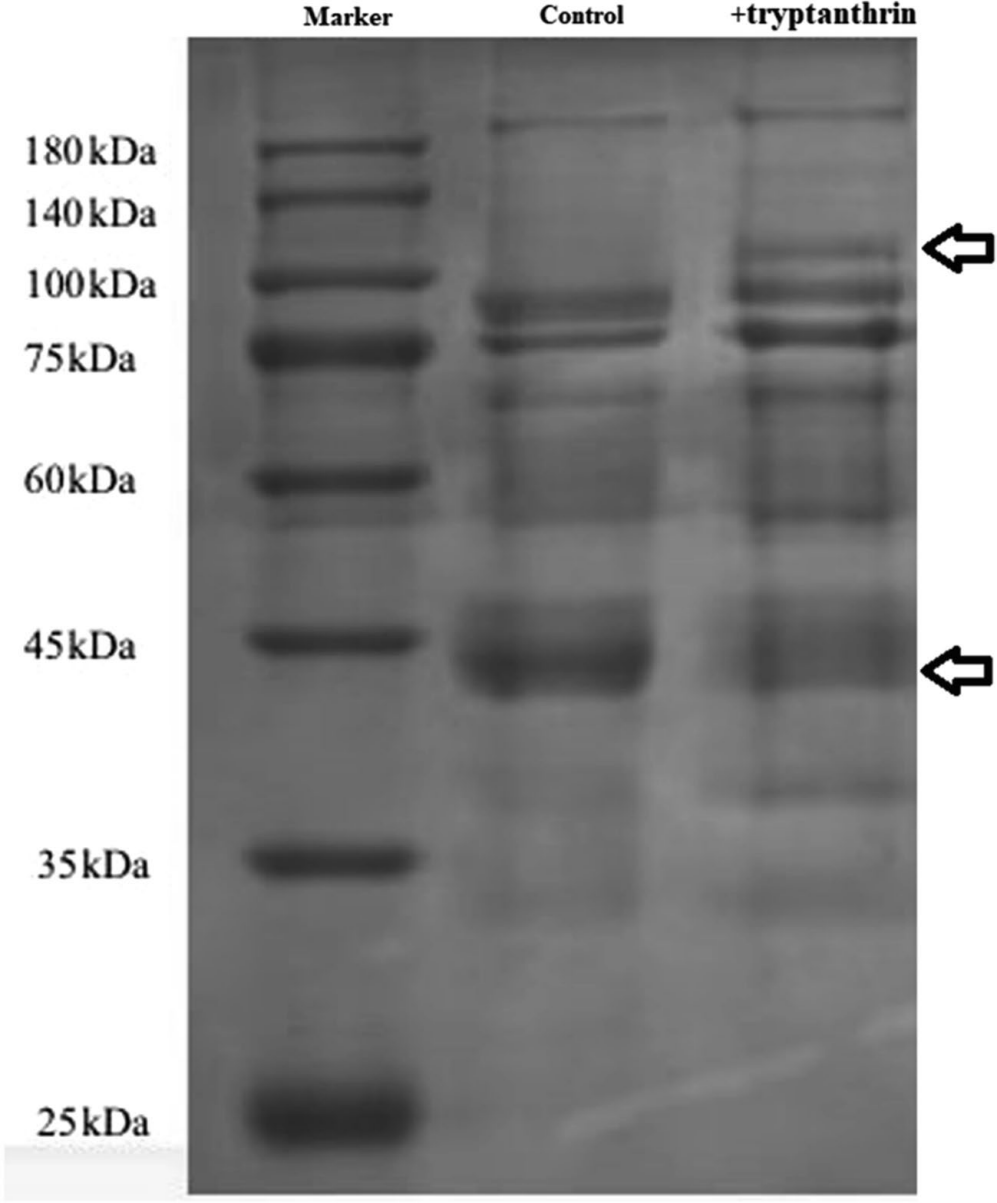
Electrophoresis of the extracellular proteins of the cells grown with and without tryptanthrin using SDS**–**PAGE. The bands of DEPs in the two samples were arrow marked

To explore whether tryptanthrin could affect the protein secretion of *V. splendidus* AJ01, supernatants collected from cultures grown with or without tryptanthrin were analyzed using SDS**–**PAGE. Distinctly different bands can be clearly observed on the gel. One band with a molecular mass of more than 100 kDa was significantly upregulated, whereas the other band at approximately 45 kDa was significantly downregulated (Fig. 3**A**). Both bands were collected, and the proteins in the bands were further determined by LC**–**MS. The results showed that the differentially expressed protein 1 (ZP_1_) was a protease, and the differentially expressed protein 2 (ZP_2_) was a flagellin protein involved in flagellar formation (Table 2).

**Table 2:** LC–MS analysis of the DEPs

Sample	Accession	Gene	MW (kD)	Description
ZP_1_	A0A2T5F0H1	CWO07_02750	101.421	An immune inhibitor with a peptidase_M6 domain
ZP_2_	A0A1A6LG25	A9262_15710	40.18	Flagellin

### Tryptanthrin affected the mRNA levels of virulence-related genes

**Fig. 4:**
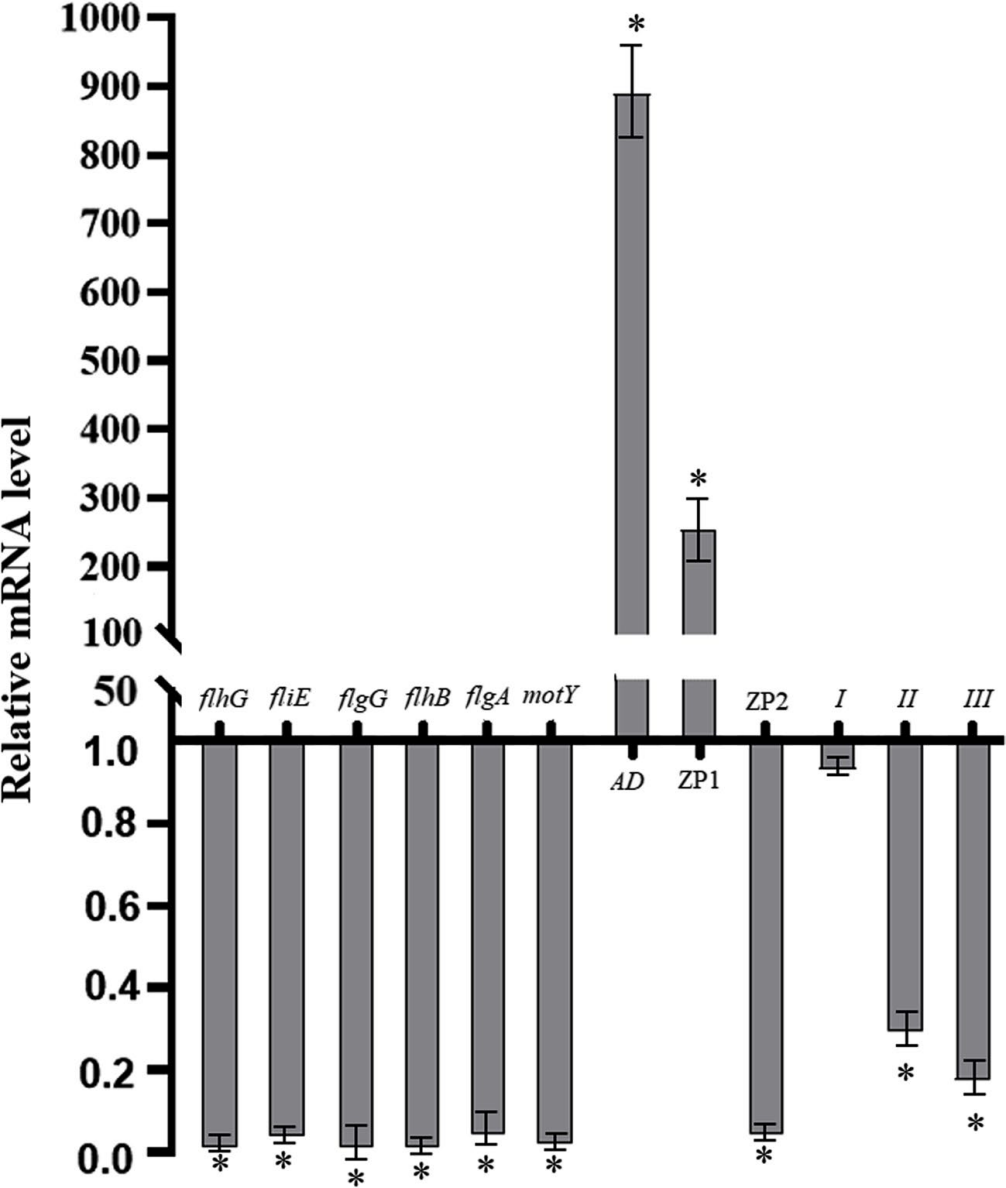
mRNA levels of the specific gene. *V. splendidus* AJ01 were cultured with or without tryptanthrin to an OD_600_ of approximately 0.4. Real-time RT**–**PCR was performed following RNA extraction and reverse transcription. 16S rRNA was used as a reference gene. The expression of the genes in the absence of tryptanthrin was defined as “1.” The average value from at least three experiments was presented as the average ± SE. *P < 0.05

Virulence-related genes, including genes related to flagellum, biofilm formation, and hemolysin, were chosen for real-time RT**–**PCR to detect the gene expression levels in cells with and without tryptanthrin. The mRNA levels of each gene in the presence of tryptanthrin are shown in Fig. 4, and the expression of each gene without tryptanthrin was used as 100%. Most of the genes involved in flagellar formation, i.e., *flhG*, *fliE*, *flhB*, *flgG*, *flgA*, and *motY*, were downregulated. Moreover, the mRNA expression of ZP_2_ was also downregulated. However, the expression levels of genes related to adhesion factor involved in biofilm formation in the cells with tryptanthrin increased. The mRNA expression of ZP_1_ protease was upregulated, exhibiting the same expression trend as the protein level in SDS**–**PAGE analysis.

### Sigma-54-dependent transcriptional regulators are the potential target of tryptanthrin in *V. splendidus*

#### Cloning and characterization of two putative LuxO_Vs_

**Fig. 5:**
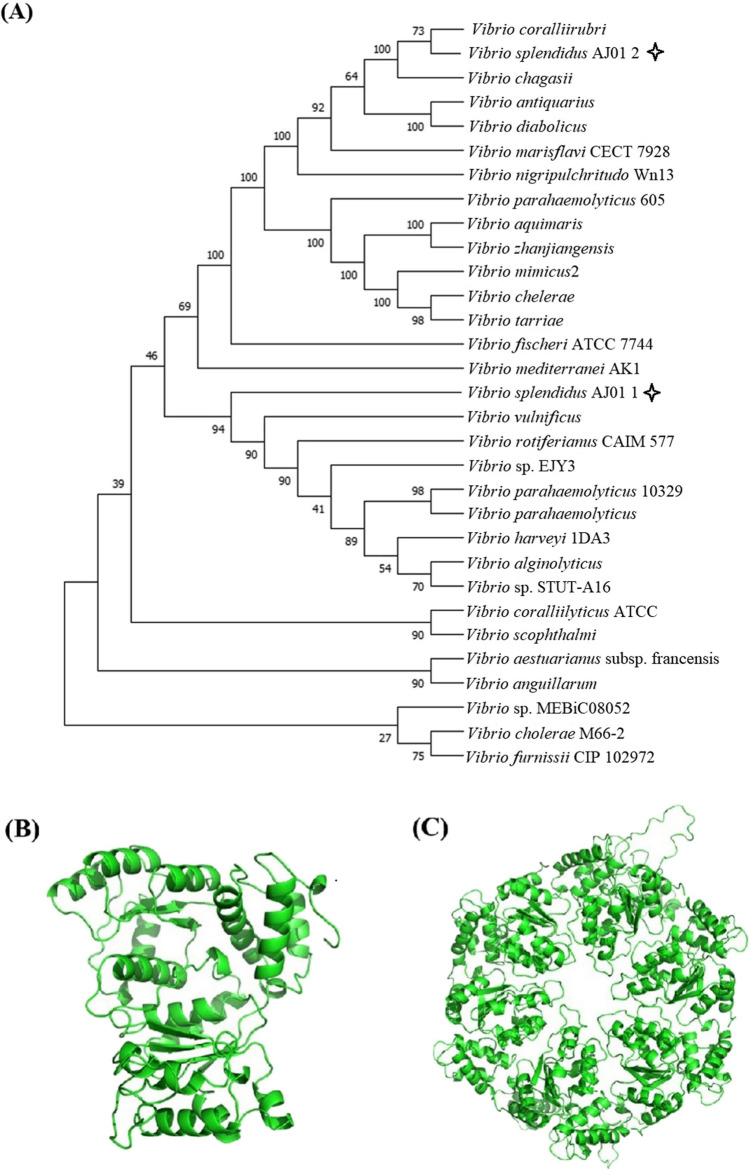
**A** Molecular evolutionary tree constructed by the NJ method using MEGA software, based on the amino acid sequences of LuxOs from different *Vibrio* species. All protein sequences are obtained from the NCBI. The sequence numbers from top to bottom are as follows: CDT55873.1, LuxO_2_, CAH7011493.1, ACY51693.1, WP_268853584.1, CAH0536155, CCO53396, ETT11540, WP_152429975.1, GLT18841, WP_334511995, WP_156845775.1, RBM47670, BAF43696.1, EDL52722, LuxO_1_, WP_332234102, PIB16600, AEX22661, EGF45372, AEL22994, EEZ87274, ABF50762.1, BDR17646, EEX31766.1, ANS84831, CAH8199495.1, WP_026028983.1, ACP05294.1, WP_059120319, and EEX42264. **B** The structure of LuxO_1_ modeled using SWISS. **C** The structure of LuxO_2_ modeled using SWISS

**Fig. 6:**
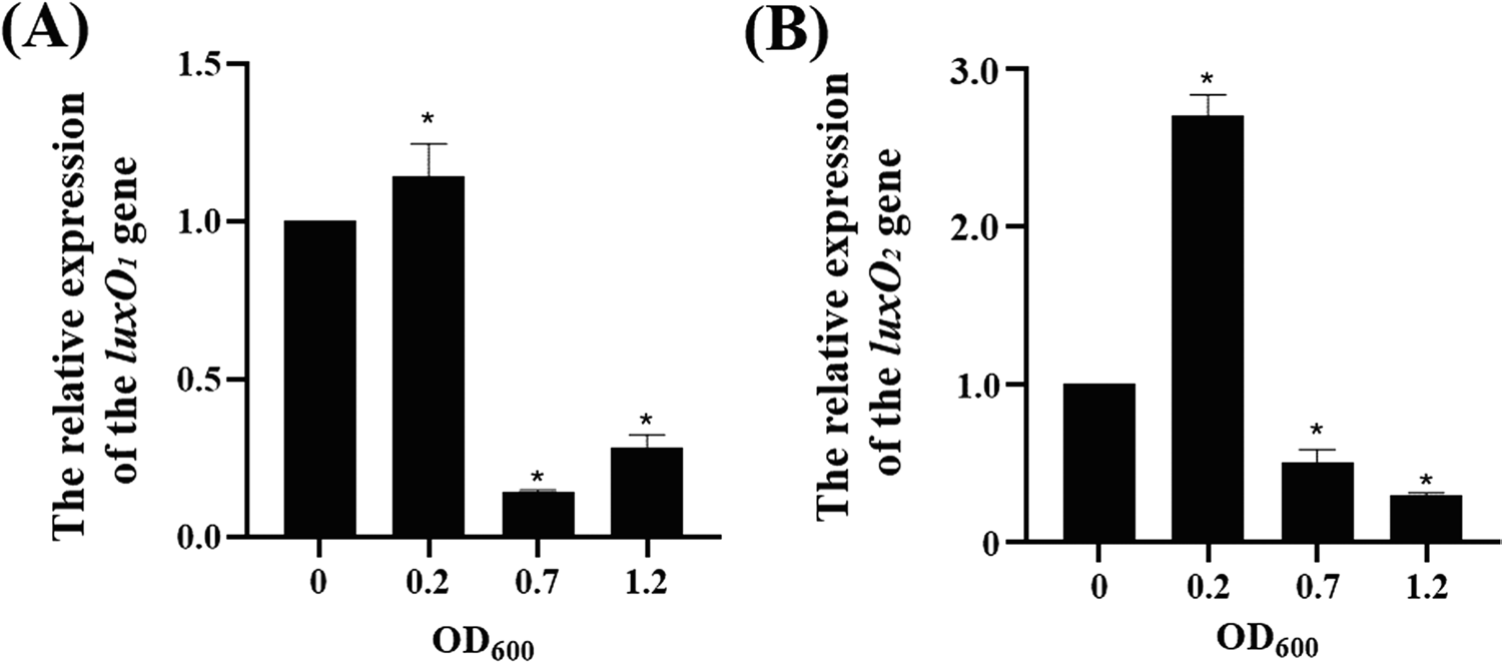
mRNA level of *luxO*_*1*_ (**A**) and *luxO*_*2*_ (**B**) in the cells at different cell densities. *V. splendidus* AJ01 was cultured to OD_600_ of 0.2, 0.7, and 1.2, respectively. Real-time RT**–**PCR was performed following RNA extraction and reverse transcription. 16S rRNA was used as a reference gene. The expression of both *luxO*s in the cells at the first of inoculation was defined as “1.” The average value from at least three experiments was presented as the average ± SE. *P < 0.05

The genomic DNA of *V. splendidus* AJ01 has two annotated *luxO* genes (unpublished data), named *luxO*_*1*_ and *luxO*_*2*_. The open-reading frame of the *luxO*_*1*_ gene was 1347 bp, and it codes LuxO_1_ with a pI of 5.8 and a molecular mass of 51.5 kDa. The open-reading frame of the *luxO*_*2*_ gene was 1512 bp, and it codes LuxO_2_ with a pI of 5.89 and a molecular mass of 56.5 kDa. Phylogenetic analysis showed that the LuxO_1_ and LuxO_2_ in *V. splendidus* AJ01 had high homology to the LuxOs of other *Vibrio* spp. (Fig. 5**A**). Based on the result of BLAST in NCBI, both LuxOs are the regulators of NtrC homolog, which are sigma-54-dependent transcriptional regulators based on the result of BLAST in NCBI, with the same modular architectures predicted using the SMART tools (Supplementary Data Fig. S1). However, the BLAST result of LuxO_1_ indicated that it was more likely a quorum sensing sigma-54-dependent transcriptional regulator. The protein structure of LuxO_1_ and LuxO_2_ in *V. splendidu*s predicted by SWISS is shown in Fig. 5**B**. Both showed binding capacity to the substrate of ATP (Supplementary Data Fig. S2 and Table S1). Two pairs of specific primers that corresponded to *luxO*_*1*_ and *luxO*_*2*_ were synthesized in accordance with the inconsistent DNA sequences of both genes, and further RT**–**PCR showed that the expression levels of both genes responded to cell densities. Their expression levels were relatively higher in *V. splendidus* AJ01 cells with lower OD_600_ than those in *V. splendidus* AJ01 cells with higher OD_600_. However, a slight difference was observed in the detailed expression profile, in which the mRNA level of *luxO*_*1*_ was consistent at lower cell density, whereas that of *luxO*_*2*_ was initially upregulated from the start to the maximum level and then decreased (Fig. 6).

#### Molecular docking of two putative LuxO_Vs_ and tryptanthrin

**Fig. 7:**
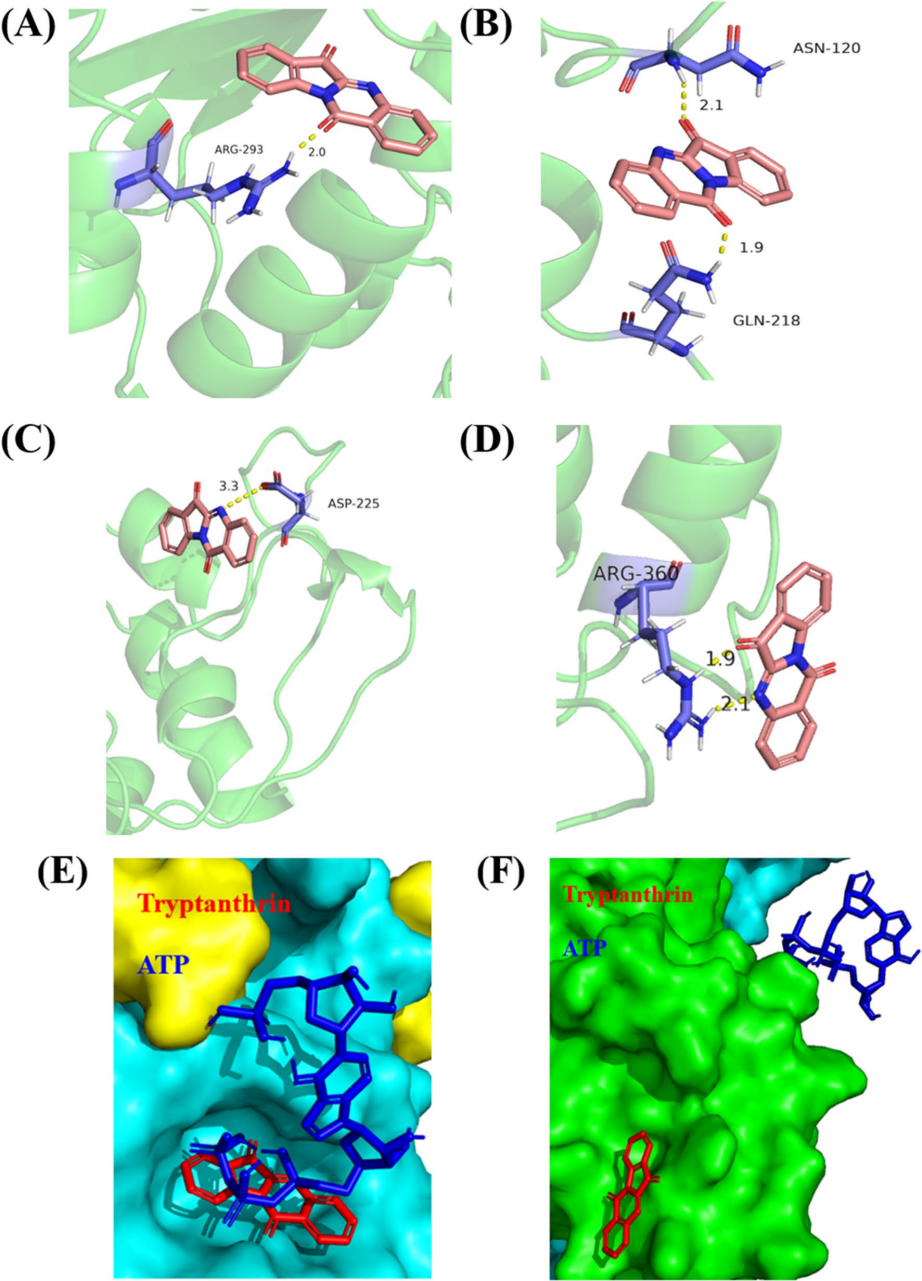
Predicted interaction of tryptanthrin with the LuxO_1_ and LuxO_2_ of *V. splendidus* AJ01 using the AutoDock software. **A** One representative prediction of LuxO_1_ interacting with tryptanthrin through a hydrogen bond. **B** One representative prediction of LuxO_1_ interacting with tryptanthrin through two hydrogen bonds. **C** One representative prediction of LuxO_2_ interacting with tryptanthrin through a hydrogen bond. **D** One representative prediction of LuxO_2_ interacting with tryptanthrin through two hydrogen bonds. **E** Prediction of the simultaneous binding of ATP and tryptanthrin to LuxO_1_.** F** Prediction of the simultaneous binding of ATP and tryptanthrin to LuxO_2_

The amino acid residues of LuxO and tryptanthrin bound through hydrophobic interactions or the benzene of amino acid, and the heterocycles of tryptanthrin formed P-P stacking interactions. In the present study, tryptanthrin could bind to LuxO_1_ and LuxO_2_, but the predicted binding sites in the two cases were different. Tryptanthrin bound to the pocket structure of LuxO_1_ by hydrogen bonding, through forming single hydrogen bonds with Arg293, Gln218, Ser121, Val69, Thr146, Asn120, or Lys195, respectively (Fig. 7**A** and **B,** Supplementary Data Table S2), while it bound to LuxO_2_ through forming single hydrogen bonds with the amino acids at amino acids of Lys253, Asp191, Arg360, Lys338, Phe194, Lys227, Arg119, or Thr348, respectively (Fig. 7**C, D;** Supplementary Data Table S2), significantly different from the binding to LuxO_1_. The model prediction showed that ATP and tryptanthrin possibly bind to the same amino acids of LuxO_1_, i.e., Arg293 or Gln218, but they could bind to different amino acids of LuxO_2_ (Fig. 7**E**, **F**). This result revealed that tryptanthrin could clearly serve as a competitive inhibitor of ATPase in LuxO_1_.

## Discussion

The environmental problems caused by residual antibiotics in the environment have led to the exploration of antibiotic alternatives for treating bacterial infection (Polianciuc et al. 2020). Antibiotic alternatives have been reported to reduce mortality caused by several marine pathogens, including *Vibrio* spp. (Yilmaz et al. 2022). Tryptanthrin is a natural phytochemical that can be used as a bacterial inhibitor (Kawakami et al. 2011), with the merits of easy availability and high safety, which facilitate its application (Kaur et al. 2017; Narendrakumar et al. 2019). Here, the antibacterial activity of tryptanthrin was explored. Tryptanthrin has been reported to possess antibacterial activity against *Mycobacterium tuberculosis* (Mitscher and Baker 1998), *Bacillus subtilis* (Fickenscher and Zähner 1971), and *E. coli* (Bandekar et al. 2010). Therefore, the present study is the first attempt to explore the inhibitory effect of tryptanthrin on the growth of marine isolates of *Vibrio* sp. as a new antibacterial agent with an MIC of 10 μg/mL, thereby widening the antibacterial spectrum of tryptanthrin. Similar to its inhibition on the growth of *E. coli* (Bandekar et al. 2010), tryptanthrin could also inhibit the biomass of *V. splendidus*, which facilitates its application in aquaculture.

*V. cholerae* forms biofilm at low cell density to survive the acidic condition under gastric environment (Rothenbacher and Zhu 2014). However, in *V. splendidus*, the biofilm is increased when there were more cells in the culture (Yang et al. 2023). In the presence of tryptanthrin, the growth was inhibited, but the biofilm formation increased. Considering the nature of the biofilm (Mah and O’Toole 2001), the increased biofilm cells suggested that the remaining cells survived the killing effect of tryptanthrin might through the biofilm formation. This was supported by the significant upregulation of one gene coding an inner membrane complex protein involved in cell attachment. Studies have shown that flagellar movement plays an important role in the formation of biofilm in the life cycle of *Vibrio* spp. (Echazarreta and Klose 2019; Teschler et al. 2015), and the initial step of biofilm development is generally accepted to be quick adherence of non-swimming cells (Belas 2013). In the present study, effects of tryptanthrin on the biofilm formation and cell motility were the opposite, which was similar to the effect of c-di-GMP on this two bacterial behaviors (Wolfe and Visick 2008). The swimming motility and the mRNA levels of genes that participated in flagellar formation of the remaining cells decreased, which contribute to the biofilm formation as described in *V. cholerae* and *Salmonella enterica* serovar Typhi, in which a negative relationship between motility and biofilm forming behavior was present (Guttenplan and Kearns 2013; Kalai Chelvam et al. 2014; Liu et al. 2010; Wolfe and Visick 2008). Although no significant effect was found on the extracellular protease activity in the presence of tryptanthrin, one extracellular protease determined as immune inhibitor A was upregulated. Immune inhibitor A is well studied in *Bacillus* sp., and it is involved in the breakage of host proteins as its pathogenesis (Pflughoeft et al. 2014). The upregulated immune inhibitor A in the presence of tryptanthrin indicated that tryptanthrin could mediate the virulence-related factors of *Vibrio* sp.

Little is known about the mechanism on the antimicrobial activity of tryptanthrin. Several proposed theories in different bacteria have been proposed. The inhibition mechanism of tryptanthrin was proposed to act as DNA intercalators, as confirmed in *E. coli* (Bandekar et al. 2010). In *Vibrio* sp., quorum sensing is the central regulatory system that controls collective behavior, including the biofilm formation, virulence, and biogeochemical cycling (Israel et al. 2023; Lami 2019; Milton 2006). Among the pathways, the highly conserved LuxO as the central component of the quorum sensing pathway was confirmed in *Vibrio* sp. *(*Boyaci et al. 2016*)*. In *V. cholerae*, the binding of tryptanthrin to LuxO showed antibiofilm activity (Narendrakumar et al. 2019). In the present study, two annotated LuxOs were proposed to be the potential target of tryptanthrin on the basis of bioinformatic analysis. The same binding sites of ATP and tryptanthrin to LuxO_1_ indicated direct competitive binding between the two chemicals, and the inconsistent binding of ATP and tryptanthrin to LuxO_2_ may work through the conformation affection as indicated previously (Boyaci et al. 2016). This consistence in *Vibrio* sp. could facilitate the exploration of the inhibitory mechanism of tryptanthrin. A previous study showed that the biofilm formation of *V. splendidus* was positively regulated by quorum sensing (Yang et al. 2023), which indicated that the phosphorylated LuxO negatively regulated biofilm formation at lower cell density. Tryptanthrin not only could maintain *V. splendidus* at relative lower cell density but also potentially inhibit the phosphorylation of LuxO, thus upregulating the biofilm formation.

## Supplementary Information

Below is the link to the electronic supplementary material.

Supplementary file1 (PDF 180 KB)

## Data Availability

The datasets generated and/or analyzed during the current study are available from one of the corresponding author, Weiwei Zhang, upon reasonable request.
