# Supplementary material for: Characterization of tryptanthrin as an antibacterial reagent inhibiting Vibrio splendidus
Source: Appl Microbiol Biotechnol. 2024 May 24;108:343. doi: 10.1007/s00253-024-13158-7 (PMC11126475; doi:10.1007/s00253-024-13158-7)
Supplement: Supplementary file 1 [file 253_2024_13158_MOESM1_ESM.pdf]

Applied Microbiology and Biotechnology

Supplemental Material

Characterization of tryptanthrin as an antibacterial reagent inhibiting *Vibrio splendidus*

Huirong Yang[a,b], Ya Li[a,b], Weibo Shi[a,b,], Weiwei Zhang[a,b,*]

[a] Collaborative Innovation Center for Zhejiang Marine High-efficiency and Healthy Aquaculture, Ningbo University, Ningbo 315832, PR China

[b] School of Marine Sciences, Ningbo University, Ningbo,

* Corresponding author:

Weiwei Zhang

169 Qixingnan Road,

Ningbo University,

Ningbo,

Zhejiang Province 315832, P. R. China

E-mail: zhangweiwei1@nbu.edu.cn

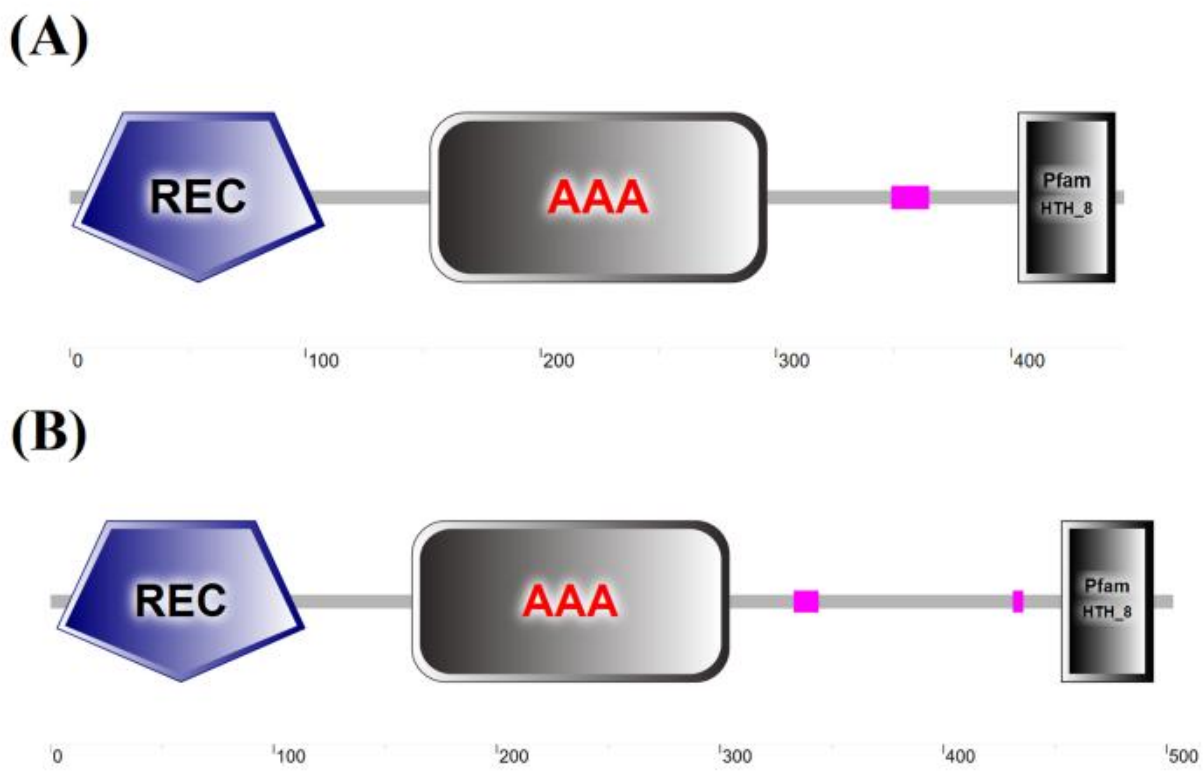

Fig. S1 The SMART analysis of $LuxO_1$ and $LuxO_2$.

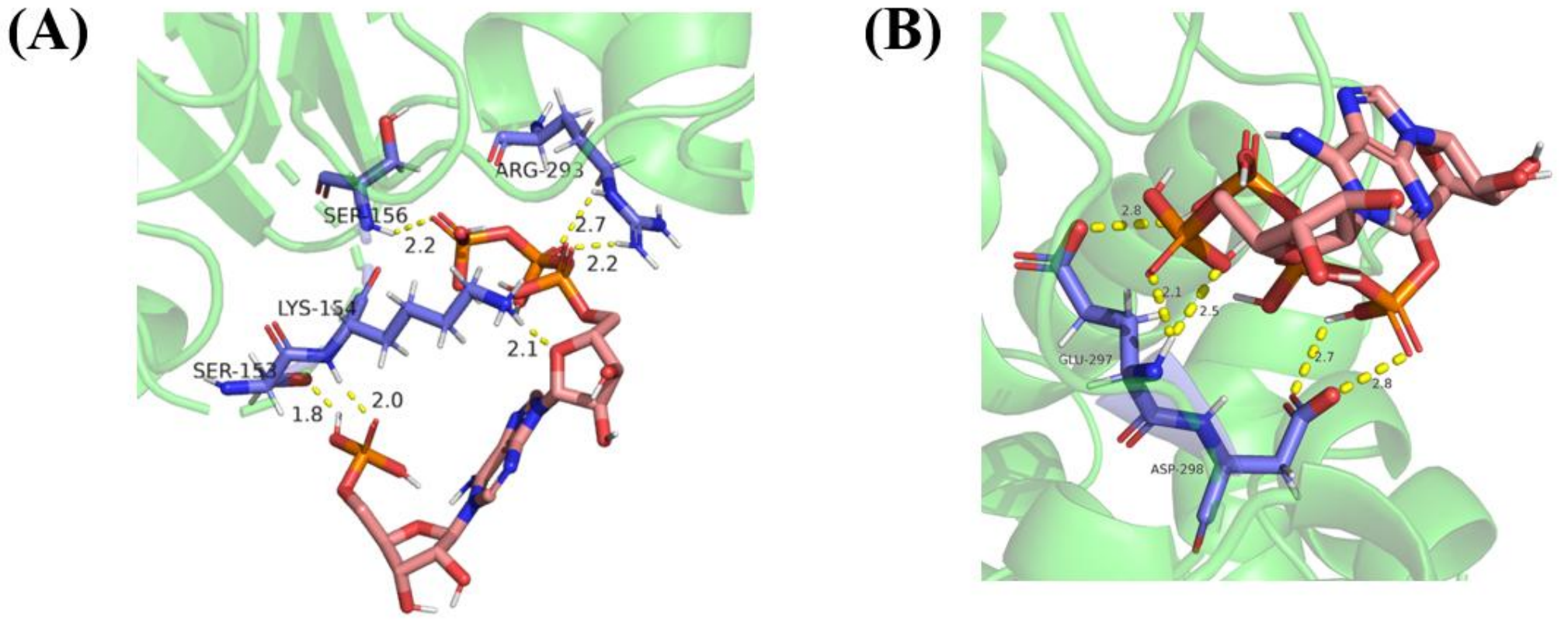

Fig. S2 One representative molecular binding prediction of $LuxO_1$ (A) and $LuxO_2$ (B) with ATP

Table S1 Prediction of the binding sites between ATP and LuxO by autodock 4. The binding sites were sorted according to the binding energy from low to high, and the lower the binding energy value, the more stable the binding ability.

	**LuxO$_1$**		**LuxO$_2$**	
	**Binding sites**	**Binding energy**	**Binding sites**	**Binding energy**
**ATP**	Lys154, Arg293	2.42	Gln297	3.83
	Gln218	4.13	Arg296	5.22
	Lys279	4.62	Arg204	5.57
	Lys179, Lys183	4.95		
	Arg217	5.03		
	Gln282, Trp278	5.33		
	Ser152, Lys179	5.46		
	Lys179	5.6		

Table S2 Prediction of the binding sites between tryptanthrin and LuxO calculated by autodock 4. The binding sites were sorted according to the binding energy from low to high, and the lower the binding energy value, the more stable the binding ability.

	**$LuxO_1$**		**$LuxO_2$**	
	**Binding sites**	**Binding energy**	**Binding sites**	**Binding energy**
	Ser121	-6.21	Lys253	-4.88
	Val69	-5.55	Asp191	-4.81
	Thr146	-5.46	Arg360	-4.77
	Lys195	-5.12	Lys338	-4.66
	Ala101	-4.39	Phe194	-4.51
	Arg293	-4.88	Lys227	-3.97
	Glu288	-4.88	Arg119	-3.92
	Arg88	-4.81	Arg2	-3.77
	Leu118	-4.63	Gln131	-3.49
	Leu288	-4.59	Lys154	-3.46
**Tryptanthrin**	Asn120, Gln218	-4.58	Thr348	-3.27
	Gln218	-4.55		
	Lys207, Lys261	-4.36		
	Asn364	-4.29		
	Lys119	-4.28		
	Asp22	-4.23		
	Tyr316	-4.12		
	Arg105	-4.02		
	Leu239	-3.96		
	Asn364	-3.92		
	Lys195	-3.8		
	Thr370	-3.71		
